# A Decade of Experience Using mTor Inhibitors in Liver Transplantation

**DOI:** 10.1155/2011/913094

**Published:** 2011-03-15

**Authors:** Jeffrey Campsen, Michael A. Zimmerman, Susan Mandell, Maria Kaplan, Igal Kam

**Affiliations:** ^1^Division of Transplant Surgery, Department of Surgery, School of Medicine, University of Colorado Denver, Aurora, CO 80045, USA; ^2^Baylor University Medical Center, Transplant, 4 Roberts, 3500 Gaston Avenue, Dallas, TX 75246, USA

## Abstract

Some studies suggest that Sirolimus (SRL) is associated with an increased risk of death in liver transplant recipients compared to treatment with calcineurin inhibitors (CNIs). We compared patients who received SRL or CNI in the first year after liver transplant. Our database included 688 patients who received a liver transplant. The patients were divided into groups. (1) CNI + MPS (mycophenolate sodium) at time of discharge. (2) CNI + MPS at time of discharge; SRL was added within the first 6 months and continued through the first year. (3) CNI + MPS at time of discharge; SRL was added within the first 6 months and discontinued before the first year. (4) SRL as primary immunosuppression. (5) SRL as primary immunosuppression and discontinued before the first year. We used mortality and graft loss as the primary measures of outcome. We also quantified renal function using the change in glomerular filtration rate (GFR), the presence of biopsy proven acute cellular reject (ACR), and steroid-resistant rejection (SRR). There were no significant differences in mortality or graft loss. There was no difference in patient or graft survival. Patients that received SRL as primary immunosuppression had 50% less rejection compared to controls.

## 1. Introduction

Sirolimus (rapamycin) is a macrolide lactone that was approved for use as an immunosuppressant in 1999 [1], but not for use in liver transplantation. It suppresses the T-cell response to interleukin-2 by binding to and inhibiting the mammalian target of rapamycin (m-TOR) [1]. There are reports of benefits and risks for the use of Sirolimus (SRL), in liver transplantation [2–17]. Investigators have reported an increase in renal failure, hepatic artery thrombosis, and overall post-transplant mortality compared to the use of calcineurin inhibitors (CNIs) [6, 11, 18]. In contrast, other studies report good outcomes and that SRL has a renal-sparing effect [9]. 

Our center has routinely used SRL for immunosuppression following liver transplantation [1, 18–26]. We have used SRL as primary therapy along with a CNI in the early post-operative period starting in January 2000 until it received a “black box” warning on the label for increased risk of hepatic artery thrombosis. After that, we converted patients to SRL therapy at various time points after discharge from the hospital. Internal review of our database showed no increase in morbidity and/or mortality in our SRL patients compared to a standard therapy of CNI+ enteric-coated mycophenolate sodium (MPS). Our preliminary data suggested that SRL can reduce donor graft rejection and could ameliorate renal injury secondary to increased use of CNI.

This study seeks to determine if there is an increase risk of complications associated with the use of SRL in the first year after liver transplantation. We collected data from all patients who had received SRL in the first year after liver transplant. Because of previously reported markers of poor outcome associated with the use of SRL, this study included overall patient mortality, donor graft loss, and renal function. Further, because our previous data suggested that SRL may decrease the incidence of rejection, we measured the rates of acute cellular rejection (ACR) and steroid resistant rejection (SRR) in our patient population [19].

## 2. Methods

### 2.1. Study Design

This study was approved by the University of Colorado Internal Review Board. We retrospectively reviewed the University of Colorado Denver transplant database and collected data on all patients who received a liver transplant between January 2000 and November 2009. This included 688 patients. An independent investigator extracted data from both electronic and paper files. Less than 3% of data was missing.

### 2.2. Immunosuppression Protocols

The pattern of immunosuppressant use allowed us to construct five study categories from the 688 patients in the database. Patients were assigned to one of the five categories according the immunosuppressive therapy given during the first year following transplantation.

The five treatment groups were.

1. Patients received a CNI + MPS at time of discharge (primary therapy) and through the first year of therapy. They never received a single dose of SRL.
2. Patients received a CNI + MPS at time of discharge; SRL was added within the first 6 months and continued through the first year.
3. Patients received a CNI + MPS at time of discharge; SRL was added within the first 6 months and discontinued before the first year.
4. SRL was started as primary immunosuppression before discharge from hospital after transplantation combined with other varying therapies including a CNI and continued for the first year.
5. SRL was started as primary immunosuppression before discharge from hospital after transplantation combined with other varying therapies including a CNI and discontinued before the first year.

The patients in group 1 were used as Controls for the study. Groups 2 and 3 patients represented Conversion Groups. The Primary Treatment Group is comprised of patients in Groups 4 and 5. Each patient was assigned to one of the five groupings. Outcomes were then compared between the five groups.

### 2.3. Primary and Secondary Outcomes of This Study

In this study, five endpoints were compared.

**Primary Endpoints**

1. Graft failure rate (time to graft failure/death).
2. Mortality rate (survival time).

**Secondary Endpoints**

1. Acute cellular rejection rate (proven by biopsy or clinical parameters).
2. Steroid resistant rejection (biopsy proven and treated with thymoglobulin or OKT3).

Graft rejection was diagnosed by liver biopsy or by elevation of liver function test in the absence of other causes of graft dysfunction. If the liver function tests were still elevated after treatment with pulse dose steroids and the patient had not received a biopsy, one was performed. ACR that was not responsive to pulse dose steroids and had biopsy proven rejection was then deemed SRR and treated with thymoglobulin or OKT3.

(3) Renal function as defined by GFR was recorded at discharge, 1 month, 6 months, and 1 year.

### 2.4. Statistical Analysis

Univariate: we computed the *P* values for graft failure (failure-free survival) and mortality (patient survival) rate comparisons using the log rank test. The event-free (“survival”) curves were computed using the Kaplan-Meier method. For graft failure, mortality, and rejection events, the event rate in any group was computed as

(1) $$\text{Event}\;\text{rate}\;=\;1000*\left(\frac{\text{total}\;\text{number}\;\text{of}\;\text{events}}{\text{total}\;\text{person}-\text{months}\;\text{followup}}\right).$$

This rate is in units of events per 1000 person-months (p-mos) of followup and is a summary statistic for the corresponding time to event curve. Rate ratios are also reported where the denominator is the rate in the reference control group and the numerator is the rate in the group being compared to the control group. 

The *P* values for comparing failure rates and mortality rates are computed using the log rank test. For rejection rate comparisons, *P* values were computed assuming an underlying Poisson process. This assumption was made (in part) since the time to each acute rejection episode was not available. Analysis of deviance results showed that the Poisson model is a good fit to the data. 

A one-way ANOVA was used to compare means for continuous variables at baseline including GFR across the five groups after confirming that a parametric model was appropriate for each on the original scale. The chi-square test/Fisher test was used to compute *P* values for comparing binary variables such as gender, hypertension, diabetes, and any particular diagnosis such as hepatocellular carcinoma (HCC) or Hepatitis C virus (HCV). The *P* value for comparing race/ethnicity was also computed using the chi-square test.

## 3. Results

### 3.1. Patient Groups and Demographics

 The five groups of patients are listed with the patient demographics listed in Table 1. The demographic features of patients in groups 2 to 5 were comparable to the Control Group 1. However, patients differed in their MELD score and warm ischemic time (WIT). Patients in groups 4 and 5 (Primary Treatment Group) had lower MELD score (18 versus 22). WIT was longer in group 4 patients compared to controls; however, this was not statistically significant. There were also fewer live donor liver transplants among group 2 patients. The Conversion Groups (2 and 3) had a lower pre-transplant GFR compared to the Control Group 1. There were fewer transplants done for HCV in Group 4 and less for ETOH in Group 3 patients.

### 3.2. Primary Endpoints

**Graft Survival**

**Figure 1:**
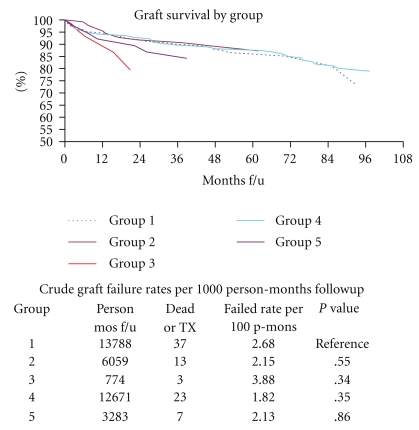
Graft survival by group. It depicts plots and tables of graft survival across the five groups represented by failure rates per 1000 person-months followup.

Figure 1 depicts plots and tables of graft survival across the five groups represented by failure rates per 1000 person-months followup. There was no statistical difference in graft survival among the five groups of patients.

**Patient Survival**

**Figure 2:**
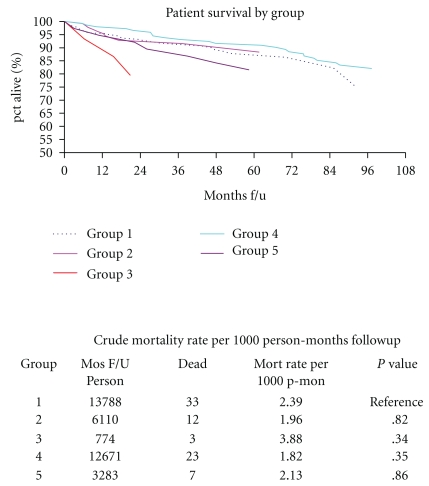
Patient survival by group. It depicts plots and tables of patient survival across the five patient categories represented by failure rates per 1000 person-months followup.

Figure 2 depicts plots and tables of patient survival across the five patient groups represented by failure rates per 1000 person-months follow up. There was no statistical difference in patient survival.

### 3.3. Secondary Endpoints

**Acute Cellular Rejection**

Table 2 depicts ACR rates per 1000 person-months of followup. ACR rates in the Groups 4 and 5 (Primary Treatment Group) where SRL was started before discharge (*P*  value ≤ .0001 and .0007) are about 50% less than the Control Group 1 and patients in the Conversion Group.

**Steroid Resistant Rejection**

Table 3 depicts SSR rates per 1000 person-months of followup and reflects the results from ACR. The incidence of ACR in the Primary Treatment Groups (4 and 5) (*P*  value = .0004  and  .038) was about 50% less than the Control Group 1.

Since ACR and SRR rates show clinically and statistically significant differences, the adjusted rates were also computed using Poisson regression as above with all 20 covariates included in the model. The 20 covariates were: recipient age, MELD score, pre-transplant GFR, BMI, donor age, CIT, WIT, male gender, ethnicity, cadaveric donor, pre-transplant diabetes, pre-transplant hypertension, HCC, HCV, PSC, Laennec's cirrhosis, HBV, AIH, NASH, and PBC. The adjusted rate ratios are similar to the unadjusted rate ratios showing that the Primary Treatment Group had about half the rejection rate compared to the Control Group.

**Glomerular Filtration Rate**

**Figure 3:**
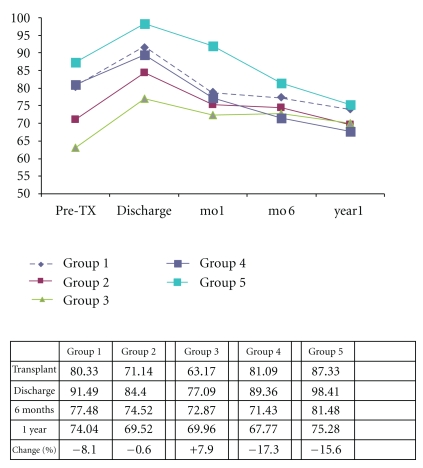
GFR (mL/min) outcome (mean). It depicts the glomerular filtration rate (GFR) (mL/min) for the five categories of patients at the times of hospital discharge, 6 months, and 1 year.

Figure 3 depicts the glomerular filtration rate (GFR) (mL/min) for the five groups of patients at the times of hospital discharge, 6 months, and 1 year. As seen in Table 2, the median pre-transplant GFR of the Conversion Groups (2 and 3) (*P*  value = .0136  and .074) was significantly less compared to the Control Group 1 at time of transplantation. In Figure 3, the percent change in GFR was calculated from pre-transplant value to the value for GFR that was obtained at 1 year after transplantation. At 6 months, patients in categories 2 and 4 had a drop in mean GFR (*P*value = .0793  and .0465) that was significantly more than the corresponding drop in controls. At one year, only group 4 had a significantly worse GRF than the Control Group with a change in GRF of −17.3% (*P*  value = .0320). While the behavior in group 5 is similar to group 4, the sample size is lower, making the *P* value larger.

At one year, 46 of the original 328 persons in the Control Group were missing, including 17 who died or were re-transplanted. We assumed that the missing patients had outcomes that were worse than those who remained in the databank. Thus, the apparent mean of 6.54 mL/min decrease in GFR in the control group could be an underestimate. The decrease in GFR is likely more than the observed 6.54 mL/min if we included those patients lost to followup. So, the one year mean GFR difference between the Control Group and the 14 mL/min drop in category 4 patients (SRL kept) is likely smaller than the results shown.

### 3.4. Tacrolimus Levels

**Figure 4:**
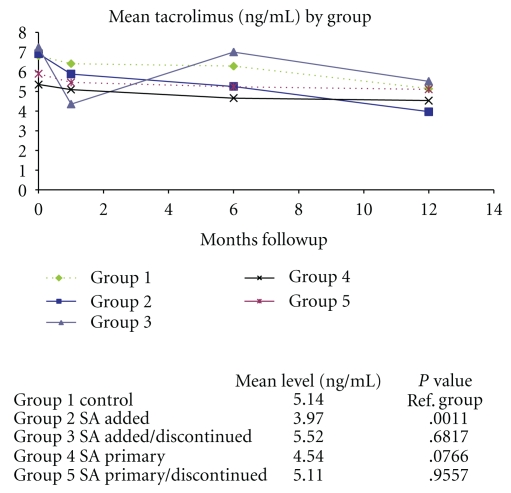
Mean Tacrolimus levels at 1 year across all five groups. It depicts mean Tacrolimus levels across all groups at 1 year.

Figure 4 depicts mean Tacrolimus levels across all groups at 1 year. Only Group 2 patients were significantly less than the Control Group 1 (*P*  value = .0011). All groups at 1 year had a mean level less than 6.0 (ng/mL).

## 4. Discussion

Our data shows that there is no increase in patient death or donor graft loss when SRL is used as primary therapy or when patients are converted to SRL following therapy with other immunosuppressants. Further, we have no evidence that suggests SRL has deleterious effects upon renal function. In contrast, our data suggests that primary therapy with SRL, but not conversion to SRL, reduced the incidence of acute and steroid resistant rejection in transplant recipients. This may be an effect of increased immunosuppression.

Sirolimus has been widely used in renal transplantation but is not currently approved for use in liver transplantation. There are reports that SRL has negative effects on outcome including HAT, delayed wound healing, and increased mortality compared to standard immunotherapy [14, 15]. Our center has used SRL therapy after liver transplantation at a number of different time points. Few other centers have this large experience using SRL in liver transplantation coupled with a nearly complete data set. Data from our previous publications did not support claims that the use of SRL is associated with negative outcomes [1, 18, 19, 22–26]. Rather, our previous data suggested that mTor inhibitors are safe when used as single therapy or in addition to CNI after liver transplant. We observed this when SRL was used as a primary immunotherapy or if SRL was added later. 

We administered SRL to patients in two regiments: we gave SRL immediately after liver transplant or added/substituted SRL within the first year following transplantation. There was a range of reasons for different combinations of therapies over the first year mostly from evolving protocols. The Conversion and Primary Treatment Groups represents a more heterogeneous collection of immunotherapies and must be interpreted with caution. However, comparison between the categories of patients within both groups did not show significant differences. We did not find differences between patient and/or donor graft survival between our patients who received SRL compared to those who received CNI + MPS. Similarly, there were no differences in patient and graft outcome that were related to whether SRL was primary or conversion therapy. 

Our secondary measures of outcome were graft rejection. Patients that received SRL as primary immunosuppression, defined as SRL administered before discharge from the hospital after liver transplant, had less ACR and SRR rejection. This was true for patients that stayed on SRL for one year and for patients that stopped using SRL before one year. This finding was statistically significant, and the rates of rejection were 50% less than the control of CNI + MPS.

Finally, we reviewed our data to see if there is a difference in renal function when SRL is used. It is well understood that CNI can cause progressive kidney injury. SRL has been used to reduce the level of CNI, thus, protecting the kidney [5]. Our data did not support this theory. All five groups had a reduction in GFR after transplant. We believe there are two reasons why our study does not support previous publications. First, since some patients die or are re-transplanted before 6 months or 1 year, their corresponding missing GFR values create a survival biases. Thus, the estimates of mean GFR at one year in particular may be biased because sicker patients that have a high mortality and also a greater reduction in GFR. Second, as seen in Figure 3, our center keeps the Tacrolimus levels in all groups at one year less than six. Thus, the lower levels of CNI may independently ameliorate the negative effects upon renal function.

We suggest that these data be interpreted with caution. First, it is a retrospective study and the Conversion and Primary Treatment Groups contain a heterogeneous group of practices. However, this data is almost 100% complete and accurately describes our practice patterns and subsequent outcome when using SRL in a variety of combinations during the first year. Secondly, because it is retrospective and we only reviewed our practice patterns for the first year, our groups are not set up to adequately assess long-term renal outcome. Rather, we can only predict kidney function within the first year of transplantation. Finally, we do not routinely perform protocol biopsies to diagnose ACR, so we could have overestimated the actual incidence of ACR in our patient population. However, the same diagnostic criteria were applied to all categories of patients that we studied. Therefore, it is unlikely that our diagnostic approach favored the diagnosis of ACR in one category of patient compared to another.

In conclusion, our goals for this study were to determine if our use of SRL during the first year after liver transplant increased mortality or morbidity. It did not. Surprisingly, SRL use as a primary therapy decreased our rates of both ACR and SRR. Based on this review, we will continue to use SRL in liver transplant recipients. With the introduction of new mTor inhibitors, such as everolimus, improved immunosuppression combinations may be developed based on our successful use of SRL.

## Figures and Tables

**Table 1:** Recipient demographics.

Variable	Group 1	Group 2	Group 3	Group 4	Group 5
*n*	328	135	15	156	38
Age (median years)	52	52	50	50	50
MELD (median)	22	22	24	18**	18
Gender male (%)	68.3	63	80	61.5	81.6
Donor age (median years)	32	30	35	30.5	33
CIT (median minutes)	352.5	373	342	334	279
WIT (median minutes)	33.5	32	36	36	38.5
Live donor (%)	15.2	8.1*	13.3	20.5	28.9
Ethnicity (%)					
Caucasian	74.4	74.07	73.3	76.3	73.3
African American	1.8	0.00	0.00	1.9	0.00
Hispanic	19.21	18.52	20.00	17.31	23.68
Asian	4.57	6.67	6.67	4.49	0.00
American Indian	0.00	0.74	0.00	0.00	2.63
BMI (median)	25.4	25.7	28.1	25.9	26.3
Pre-GFR (median)	80.4	66.8	60.3	82.7	88.7
Primary transplant indication (%)					
HCC	30.8	26.7	33.3	19.9	23.7
HCV	53.7	39.3	53.3	38.5*	50.0
PSC	11.3	14.1	0.0	14.1	13.2
ETOH	7.0	10.4	26.7	12.2	7.9
HBV	6.1	5.9	13.3	8.3	5.3
AIH	4.3	8.1	0.0	7.1	10.5
NASH	1.5	2.2	6.7	3.2	0.0
PBC	3.7	1.5	0.0	7.1	2.6
Cryptogenic cirrhosis	7.6	8.1	0.0	6.4	5.3
Budd-Chari	1.2	2.2	0.0	0.6	5.3

Statistically significant values are bolded: *P < .01, **P < .001.

**Table 2:** Acute cellular rejection rates per 1000 person-months.

Group	Person mos f/u	Rate per 1000 p-mon	*P* value
1	13788	12.5	Reference
2	6059	13.4	.64
3	774	12.9	.93
4	12671	6.5	<.001
5	3242	5.2	<.001

**Table 3:** Steroid-resistant rejection rates per 1000 person-months.

Group	Person mos f/u	Rate per 1000 p-mon	*P* value
1	13788	3.48	reference
2	6059	4.29	.03
3	774	3.88	.83
4	12671	1.58	<.001
5	3242	1.54	.04

## Abbreviations

ACR: Acute cellular reject
CNI: Calcineurin inhibitors
GFR: Glomerular filtration rate
m-TOR: Rapamycin
MPS: Mycophenolate sodium
SRL: Sirolimus
SRR: Steroid-resistant rejection.
